# Isoaspartate Accumulation in Mouse Brain Is Associated with Altered Patterns of Protein Phosphorylation and Acetylation, Some of Which Are Highly Sex-Dependent

**DOI:** 10.1371/journal.pone.0080758

**Published:** 2013-11-05

**Authors:** Zhenxia Qin, Rachel S. Kaufman, Rana N. Khoury, Mitri K. Khoury, Dana W. Aswad

**Affiliations:** Department of Molecular Biology & Biochemistry, University of California Irvine, Irvine, California, United States of America; University of Iowa, United States of America

## Abstract

Isoaspartate (isoAsp) formation is a major source of protein damage that is kept in check by the repair function of protein L-isoaspartyl methyltransferase (PIMT). Mice deficient in PIMT accumulate isoAsp-containing proteins, resulting in cognitive deficits, abnormal neuronal physiology and cytoarchitecture, and fatal epileptic seizures 30–60 days after birth. Synapsins I and II, dynamin-1, collapsin response mediator protein 2 (CRMP2), and α/β-tubulin are major targets of PIMT in brain. To investigate links between isoAsp accumulation and the neurological phenotype of the KO mice, we used Western blotting to compare patterns of *in vivo* phosphorylation or acetylation of the major PIMT targets listed above. Phosphorylations of synapsins I and II at Ser-9 were increased in female KO vs. WT mice, and acetylation of tubulin at Lys-40 was decreased in male KO vs. WT mice. Average levels of dynamin-1 phosphorylation at Ser-778 and Ser-795 were higher in male KO vs. WT mice, but the statistical significance (P>0.1) was low. No changes in phosphorylation were found in synapsins I and II at Ser-603, in CRMP2 at Ser-522 or Thr-514, in DARPP-32 at Thr-34, or in PDK1 at Ser-241. General levels of phosphorylation assessed with Pro-Q Diamond stain, or an anti-phosphotyrosine antibody, appeared similar in the WT and KO mice. We conclude that isoAsp accumulation is associated with altered functional status of several neuronal proteins that are highly susceptible to this type of damage. We also uncovered unexpected differences in how male and female mice respond to isoAsp accumulation in the brain.

## Introduction

 Isoaspartate (isoAsp) formation, through deamidation of asparaginyl residues or isomerization of aspartyl residues, constitutes a major source of spontaneous protein damage occurring *in vivo* and *in vitro* [1-7]. Generation of isoAsp sites is initiated by nucleophilic attack on the Asx side-chain carbonyl by the C-flanking amide bond nitrogen resulting in an intermediate succinimide, hydrolysis of which generates a mixture of α-linked L-aspartyl (~15-30%) and β-linked L-isoaspartyl (~70-85%) residues. Synthetic peptide studies have shown that altering the N+1 residue has a major influence on the propensity for isoAsp formation, with glycine, serine, and histidine most associated with “hot spots” of isoAsp formation. In structured proteins the same sequence effect is often found, but isoAsp formation is conformation dependent and usually restricted to flexible regions of the polypeptide.

 Protein L-isoaspartate *O*-methyltransferase (PIMT; EC 2.1.1.77), selectively methylates the α-carboxyl group of L-isoaspartyl residues [8-10]. The isoAsp methyl esters produced are highly labile (T_1/2_^≈^ 7 min at pH 7.4, 37 °C for -isoAsp-Gly-) and spontaneously demethylate to reform a succinimide that can restore the normal α-linked Asp-Xaa bond [11,12]. Continuing cycles of PIMT action have been shown to efficiently repair L-isoAsp sites in a number of peptides and proteins [13-17]. A repair function for PIMT *in vivo* is supported by observations that reduction of PIMT activity in cultured cells or knockout (KO) mice dramatically increase the level of isoaspartyl proteins [18-21]. A critical need for PIMT action in the brain is indicated by its high specific activity in this tissue [22,23] as well as the overt neurological phenotype of PIMT KO mice: increased brain size with abnormal neuro-anatomical and electrophysiological properties, impaired learning, and fatal epileptic seizures beginning at 4 weeks of age [19,20,24-26].

 Recent studies utilizing the PIMT KO mouse are shedding light on mechanisms by which isoAsp accumulation interferes with neuronal function. One mechanism may be disruption of gene expression. The flexible N-terminal region of histone H2B accumulates high levels of isoAsp *in vivo*, which could potentially alter chromatin function [27,28]. Bidinosti et al. [29,30] have shown that a brain-enriched regulator of mRNA translation, 4E-BP2, is highly susceptible to deamidation and isoAsp formation *in vivo*. Isoaspartyl 4E-BP2 exhibits decreased affinity for the mTORC1 complex, resulting in decreased protein synthesis that dramatically affects neuronal properties. Stimulated by the larger brain size of PIMT-KO mice, Farrar et al. [31] investigated the expression and phosphorylation state of proteins involved in insulin-dependent growth signaling. Using rapidly excised brain tissue from KO mice, they reported increased expression of the insulin receptor β-subunit and hyperphosphorylation of several protein kinases in the PI3K/Akt signaling pathway. Kosugi et al. [21] used a PIMT knockdown strategy to raise isoAsp levels in human embryonic kidney cells. In response to stimulation by epidermal growth factor, these cells exhibited hyperphosphorylation of Raf-1, ERK1/2, and MEK, all components of the mitogen-activated protein kinase cascade that regulates gene expression.

 In 2006, our lab used a proteomic analysis of PIMT-KO mouse brain extracts to identify 22 proteins that are highly prone to isoAsp formation *in vivo* [32]. Prominent among this group were proteins involved in regulating synaptic transmission (synapsins I and II, and dynamin-1) and cytoskeletal structure (collapsin response mediator protein 2 (CRMP2/DPYSL2/ULIP2), and α/β-tubulin). All four of these proteins are regulated by reversible phosphorylation and one of them (tubulin) is also regulated by acetylation of a lysine residue. We wondered if isoAsp formation in these proteins alters their function in a manner that might contribute directly to the neurological deficits of the PIMT deficient mice. Toward this goal, we report here the use of modification-specific antibodies to monitor the effect of PIMT deficiency on site-specific phosphorylation of synapsins I and II, dynamin-1, and CRMP2, as well as acetylation of α-tubulin. In brain extracts of the PIMT KO mouse we found increased phosphorylation of synapsins I and II, decreased acetylation of α-tubulin, and a possible increase in the phosphorylation of dynamin-1. We propose that these changes in post-translational modification reflect functional deficits in the resident proteins, and that these deficits contribute to the abnormal synaptic physiology and cytoskeletal structures that are associated with the neurological phenotype of the PIMT KO mouse.

## Materials and Methods

### Reagents

Euthasol^®^ and protease inhibitor cocktail were purchased from Virbac Corporation and Sigma-Aldrich, respectively. Sources and working dilutions of the primary antibodies used in Western blots are indicated in Table 1. HRP-conjugated donkey anti-rabbit IgG secondary antibody was purchased from GE Healthcare. HRP-conjugated rabbit anti-sheep secondary antibody and Pierce ECL Plus Substrate were purchased from Thermo Scientific. HRP-conjugated goat anti-mouse secondary antibody was from Santa Cruz Biotechnology. Pro-Q Diamond phosphoprotein stain and SYPRO Ruby protein stain were purchased from Invitrogen/Life Technologies. NaF was purchased from Fisher Scientific and Na_3_VO_4_ was purchased from Sigma-Aldrich.

**Table 1:** Antibodies used for Western blotting.

**Antigen**	**Host**	**P/M^a^**	**Dilution**	**Supplier**	**Catalog no.**
pPDK1 (Ser-241)	rabbit	P	1: 1,000	Cell Signaling	3061
Synapsin I	rabbit	P	1:10,000	Millipore	AB1543P
pSynapsin I (Ser-9)	rabbit	P	1: 1,000	Cell Signaling	2311S
pSynapsin I (Ser-603)	rabbit	P	1: 8,000	Sigma-Aldrich	S8192
pDARPP-32 (Thr-34)	rabbit	M	1: 5,000	Cell Signaling	5393S
CRMP2	rabbit	P	1: 1,000	Millipore	AB9218
pCRMP2( Thr-514)	rabbit	P	1: 1,000	Cell Signaling	9397
pCRMP2 (Ser-522)	rabbit	P	1: 1,000	ECM Biosciences LLC	CP2191
Dynamin-1	mouse	M	1:8,000	Cell signaling	4565
pDynamin-1 (Ser-778)	sheep	P	1: 2,000	Thermo scientific	PA1-4621
pDynamin-1 (Ser-795)	rabbit	P	1: 2,000	Santa Cruz Biotechnology	SC-12937
Tubulin	rabbit	P	1:16,000	Cell Signaling	2148
Ac-Tubulin (Lys-40)	rabbit	P	1: 1,000	Cell Signaling	3971S
Phosphotyrosine antibody (clone 4G10)	mouse	M	1: 4,000	Millipore	05-321
PIMT	rabbit	P	1:2,500	Custom	
β-actin	rabbit	M	1:30,000	Cell Signaling	4970S

^a^ **P**olyclonal or **M**onoclonal

### Mice

PIMT +/- (HZ) males used to start our mouse colony were kindly provided by Mark Mamula (Yale University, New Haven, CT) and were originally generated by inserting a neomycin resistance cassette into exon one of the PIMT gene [19]. PIMT -/- (KO) and PIMT +/+ (WT) C57BL/6 mice were obtained by intercrossing PIMT HZ C57BL/6 mice. Genotyping from tail clips was carried out by PCR at Transnetyx, Inc. (Cordova, TN) with probes for the neo cassette and the PIMT gene. Mice were monitored by on-site veterinarians, with all protocols undertaken in strict accordance with the recommendations for the Care and Use of Laboratory Animals, and approved by the University of California at Irvine Institutional Animal Care and Use Committee. Mice were anesthetized with Euthasol^®^ and sacrificed by decapitation at an age of 4 weeks.

### Brain Extract Preparation

Brains were homogenized using a Potter-Elvejhem tissue homogenizer in 9 vol of ice-cold lysis buffer containing 5 mM K-Hepes, 0.5 mM EDTA, pH 7.6, 10% sucrose, 50 mM NaF, 1 mM Na_3_VO_4_, 0.1 mM DTT (dithiothreitol) and 1% (v/v) protease inhibitor cocktail. After centrifugation at 800 × g for 30 min at 4°C, the supernatants (hereafter "brain extracts”) were collected and protein concentration was measured using the micro-BCA assay (Thermo Scientific) against a BSA standard.

### Western Blots

Thirty microgram samples of brain extract protein were subjected to electrophoresis in 10% NuPAGE^®^ Bis-Tris gels (Invitrogen/Life Technologies) after heating at 70°C for 10 min in SDS sample buffer. After semi-dry transfer to PVDF, the membrane was blocked with 5% non-fat milk in TBS-T (Tris-buffered saline, with 0.01% (v/v) Tween-20) and phosphatase inhibitors (50 mM NaF and 1 mM Na_3_VO_4_), and then incubated with antibodies diluted as indicated (Table 1) in the same buffer. Immunoreactive bands were developed with ECL Plus according to the manufacturer's directions. The chemiluminescent signals from immunoblots were acquired as 12-bit TIFF files using a digital SLR camera with exposure times ranging from 15 s to 4 min [33]. Band densities and background were quantified using ImageJ-64 (http://rsbweb.nih.gov/ij/index.html) for Mac OSX. For each primary antibody used, the ECL response was optimized for linearity over a 32X range with regard to protein load, antibody dilution, and camera exposure time. 

### Gel Staining

 Phosphorylated and total proteins were stained in gel with the Pro-Q Diamond phosphoprotein stain and SYPRO Ruby stain, respectively, according to the manufacturers instructions. Brain extract proteins (2 µg per lane) were subjected to electrophoresis on NuPAGE 10% Bis-Tris gels. These were fixed and stained with Pro-Q Diamond and imaged on a Typhoon™ Variable Mode Imager (GE Healthcare). The same gels were then stained with SYPRO Ruby and imaged again on the Typhoon.

### Statistical Analysis

 Band densities were corrected for background and then normalized to the β-actin signal of the same lane. The mean value of the of all the WT bands on a given gel was calculated and used to normalize values of all the individual bands. After this double-normalization, paired t-tests were carried out using adjacent lanes (e.g., WT1 vs. KO1) for pairing. Pairwise analysis minimized errors due to subtle gradients of ECL background signal on the blots. Differences were considered significant at P ≤ 0.05.

## Results

### Effect of PIMT Deficiency on Phosphorylation of Synapsins I and II

The synapsins are important for regulation of neurotransmitter release [34] and are major *in vivo* substrates for PIMT [32]. Synapsin function is regulated by phosphorylation at several distinct sites, the most studied of which are Ser-9 (also known as site 1, modified by the cAMP-dependent protein kinase (PKA) and the type I calcium-dependent protein kinase (CaMKI)) and Ser–603 (also known as site 3, modified by the type II calcium-dependent protein kinase (CaMKII)). We employed Western blotting with phosphorylation-specific antibodies to see if modification of synapsin at these sites is altered in PIMT deficient mice, as this might help explain the neurological pathology of these mice. Pilot experiments, in which extracts of male and female mice were electrophoresed on the same gel, revealed increased phosphorylation in KO vs. WT brain, but unexpectedly suggested that the effect was stronger in females than males. We therefore continued the study using alternating lanes of WT *vs.* KO brain extracts from a single sex on each gel.

**Figure 1:**
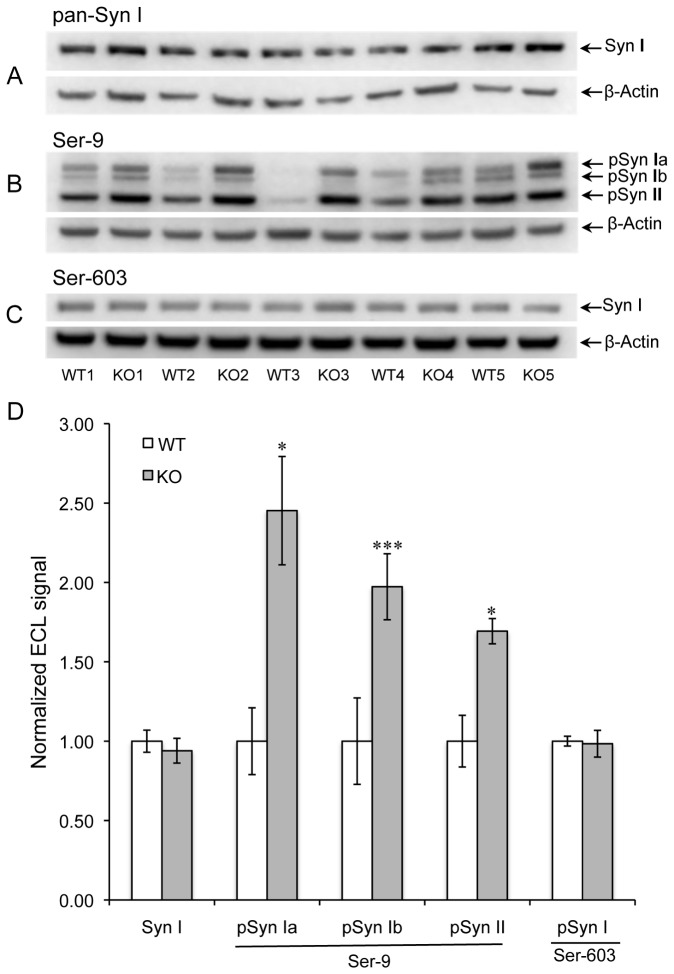
Phosphorylation of synapsins I and II in female mice. Western blot of mouse brain extracts used a mixture of antibodies to synapsin I (Syn1) and β-actin (A), a mixture of antibodies to synapsins phosphorylated at Ser-9 and β-actin (B), and a mixture of antibodies to synapsins phosphorylated at Ser-603 and β-actin (C). Panel D shows quantitative measurements of band intensities after normalization to β-actin. Data are expressed as means ± SEM (n=5 for each genotype). A two-tailed t-test with pairwise matching of adjacent lanes (WT vs. KO) was employed. *P < 0.05, **P < 0.01, ***P < 0.001 for KO vs. WT.

Results obtained from four-week female mice are shown in Figure 1 and summarized in Table 2. Western blot images obtained with antibodies made against pan-synapsin I, the phosphorylated Ser-9 site, and the phosphorylated Ser-603 site are shown in Figure 1 (A-C). The bar graph in Figure 1D summarizes these results and indicates the WT vs. KO statistical significance based on a pairwise t-test. The signal data is normalized to β-actin expression within each lane. The pSer-9 antibody used in Western blot produced signals consistent with the molecular weights of synapsins Ia, Ib and II. Phosphorylation of all three synapsin isoforms was markedly higher in the KO extracts, with average increases of 149 %, 99%, and 70% for the Ia, Ib, and II isoforms, respectively. In contrast, we observed no significant difference in synapsin I expression or in phosphorylation of synapsin I at the Ser-603 site. 

**Table 2:** Summary and statistical analysis of Western blot data.

**Antigen**	**Sex**	N^a^	**WT signal**	**WT SE** ^b^	**KO signal**	**KO SE**	**ttest** ^c^ **P**
pPDK1 (Ser-241)	F	5	1.00	0.03	0.94	0.04	0.307
	M	5	1.00	0.04	0.95	0.04	0.570
Synapsin I	F	5	1.00	0.07	0.94	0.08	0.493
**pSyn Ia (Ser-9)^d^**	**F**	**5**	**1.00**	**0.21**	**2.45**	**0.34**	**0.018**
**pSyn Ib (Ser-9)**	**F**	**5**	**1.00**	**0.27**	**1.97**	**0.21**	**<0.001**
**pSyn IIb (Ser-9)**	**F**	**5**	**1.00**	**0.16**	**1.69**	**0.08**	**0.028**
pSyn I (Ser-603)	F	5	1.00	0.03	0.98	0.08	0.832
Synapsin I	M	5	1.00	0.06	1.03	0.02	0.683
**pSyn Ia (Ser-9)**	**M**	**5**	**1.00**	**0.13**	**1.22**	**0.21**	**0.354**
**pSyn Ib (Ser-9)**	M	5	1.00	0.16	1.13	0.25	0.402
pSyn II (Ser-9)	M	5	1.00	0.08	1.13	0.09	0.318
pSyn I (Ser-603)	M	5	1.00	0.08	1.03	0.06	0.774
pDARPP-32 (Thr-34)	F	6	1.00	0.05	0.83	0.11	0.292
	M	6	1.00	0.12	1.00	0.10	0.998
CRMP2-A	F	5	1.00	0.05	0.98	0.03	0.679
CRMP2-B	F	5	1.00	0.05	1.02	0.03	0.701
pCRMP2 (Thr-514)	F	5	1.00	0.05	1.01	0.03	0.842
pCRMP2 (Ser-522)	F	6	1.00	0.05	1.03	0.07	0.713
CRMP2-A	M	5	1.00	0.05	0.98	0.04	0.667
CRMP2-B	M	5	1.00	0.04	1.05	0.07	0.428
pCRMP2 (Thr-514)	M	5	1.00	0.04	1.00	0.03	0.855
pCRMP2 (Ser-522)	M	6	1.00	0.05	0.97	0.06	0.588
Dynamin-1	F	6	1.00	0.08	1.05	0.16	0.633
pDyn1 (Ser-778)	F	6	1.00	0.19	0.96	0.33	0.902
pDyn1 (Ser-795)	F	6	1.00	0.31	0.90	0.44	0.852
Dynamin-1	M	6	1.00	0.09	1.04	0.12	0.634
**pDyn1 (Ser-778)**	**M**	**6**	**1.00**	**0.35**	**1.73**	**0.58**	**0.342**
**pDyn1 (Ser-795)**	**M**	**6**	**1.00**	**0.35**	**1.78**	**0.39**	**0.148**
Tubulin	F	5	1.00	0.04	0.96	0.02	0.486
Ac-αTub (Lys-40)	F	5	1.00	0.16	0.81	0.11	0.360
Tubulin	M	5	1.00	0.05	1.01	0.05	0.688
**Ac-αTub (Lys-40)**	**M**	**5**	**1.00**	**0.12**	**0.76**	**0.12**	**0.003**

^a^Number of animals per genotype.

^b^Standard Error

^c^Two-tailed, with matched pairing of adjacent lanes.

^d^Bold face indicates data where the KO signal is ≥20% above or below the WT signal.

**Figure 2:**
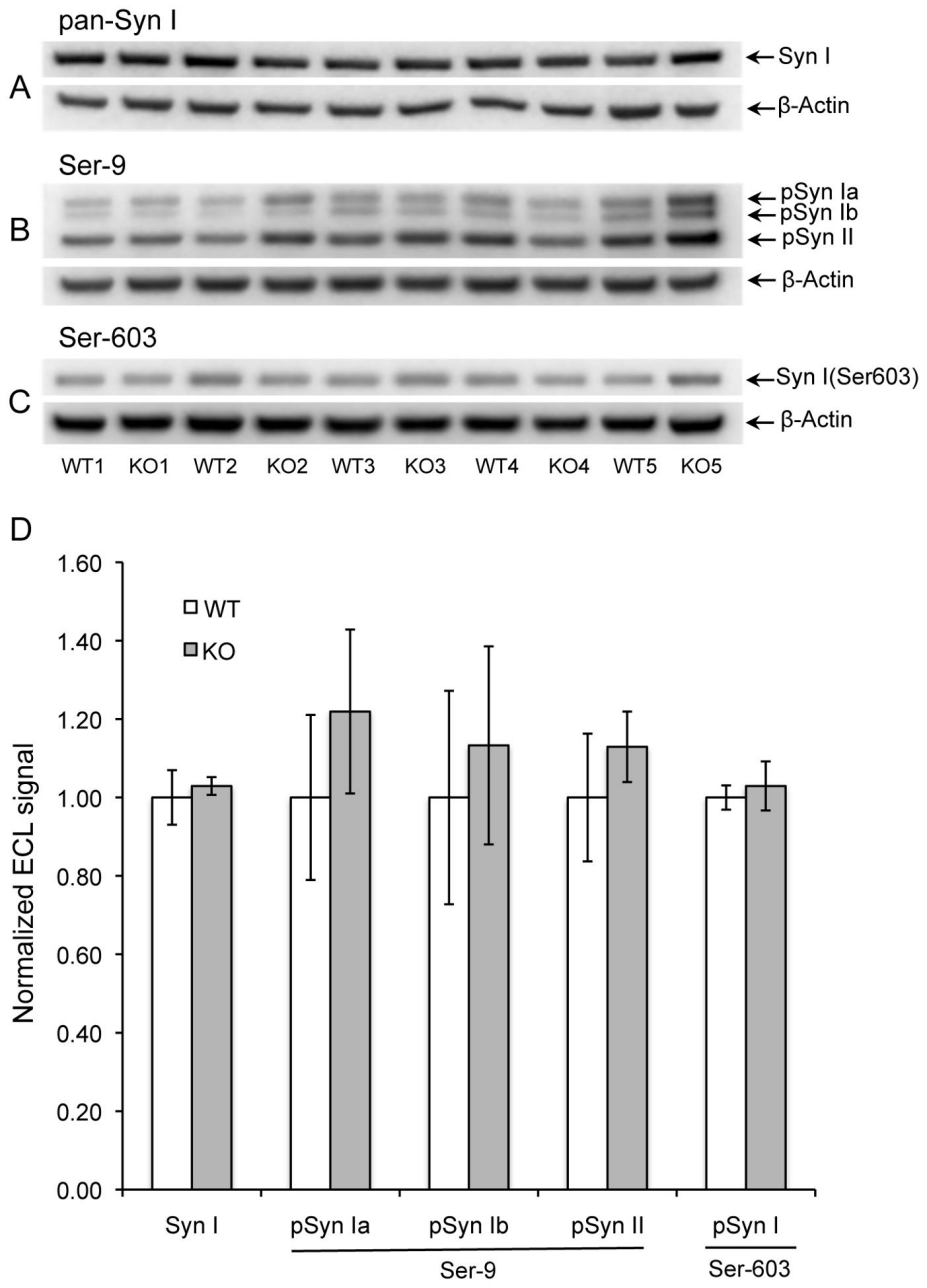
Phosphorylation of synapsins I and II in male mice. Panels A-D are otherwise the same as in Figure 1.

**Figure 3:**
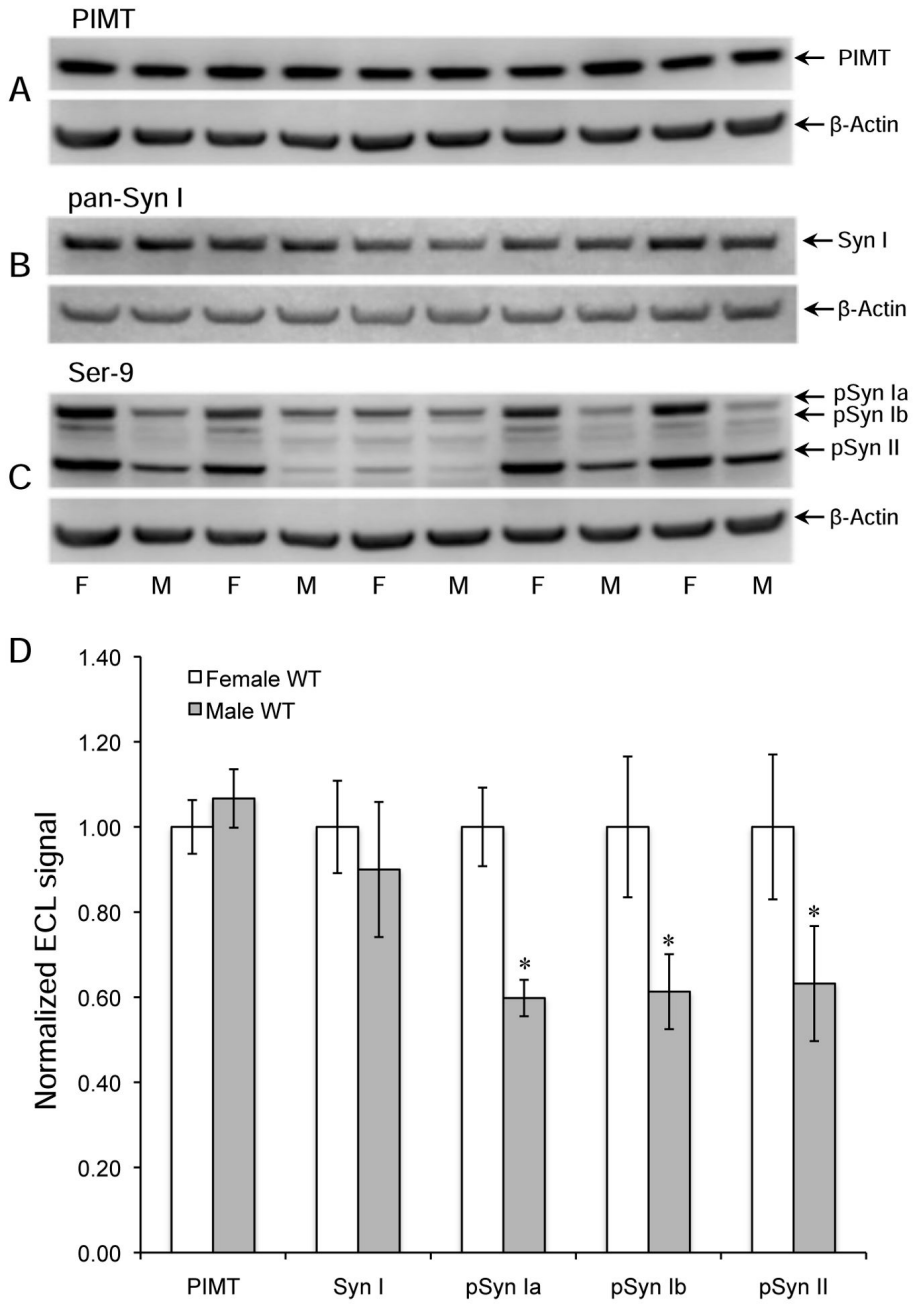
Levels of PIMT, synapsin I, and phospho-synapsin (Ser-9) in WT female and male mice. Mouse sex for each lane is indicated at the bottom on panel C. Western blots used a mixture of antibodies to PIMT and β-actin (A), a mixture of antibodies to synapsin 1 and β-actin (B), and a mixture of antibodies to synapsin 1 phosphorylated at Ser-9 and β-actin (C). Panel D shows quantitative measurements of band intensities in A-C after normalization to β-actin. Data are expressed as means ± SEM (n=5 for each sex) with statistics as in Figure 1.

Extracts from 4-week male mice showed qualitatively similar results, but the degree of enhanced phosphorylation at Ser-9 in the KO mice was much smaller; 22%, 14%, and 14%, for synapsins Ia, Ib, and II, respectively, with P > 0.05 in all three cases (Figure 2). This suggests an inherent sex difference in synapsin phosphorylation. To test this idea, we electrophoresed brain extracts of WT female and male mice in alternating lanes of the same gel prior to Western blotting for PIMT, pan-synapsin I, and pSer-9 synapsin (Figure 3A-C). As shown in Figure 3D, there was no significant sex difference in the expression of PIMT or synapsin I, yet the phosphorylation of synapsin at Ser-9 was 37- 40% lower in males than females for all three detected isoforms of synapsin. Overall these results indicate that female mice have a greater average level of phosphorylation of Ser-9 than males, and that they also show a greater increase in phosphorylation at this site in response to PIMT deficiency.

### Phosphorylation of Dynamin-1

**Figure 4:**
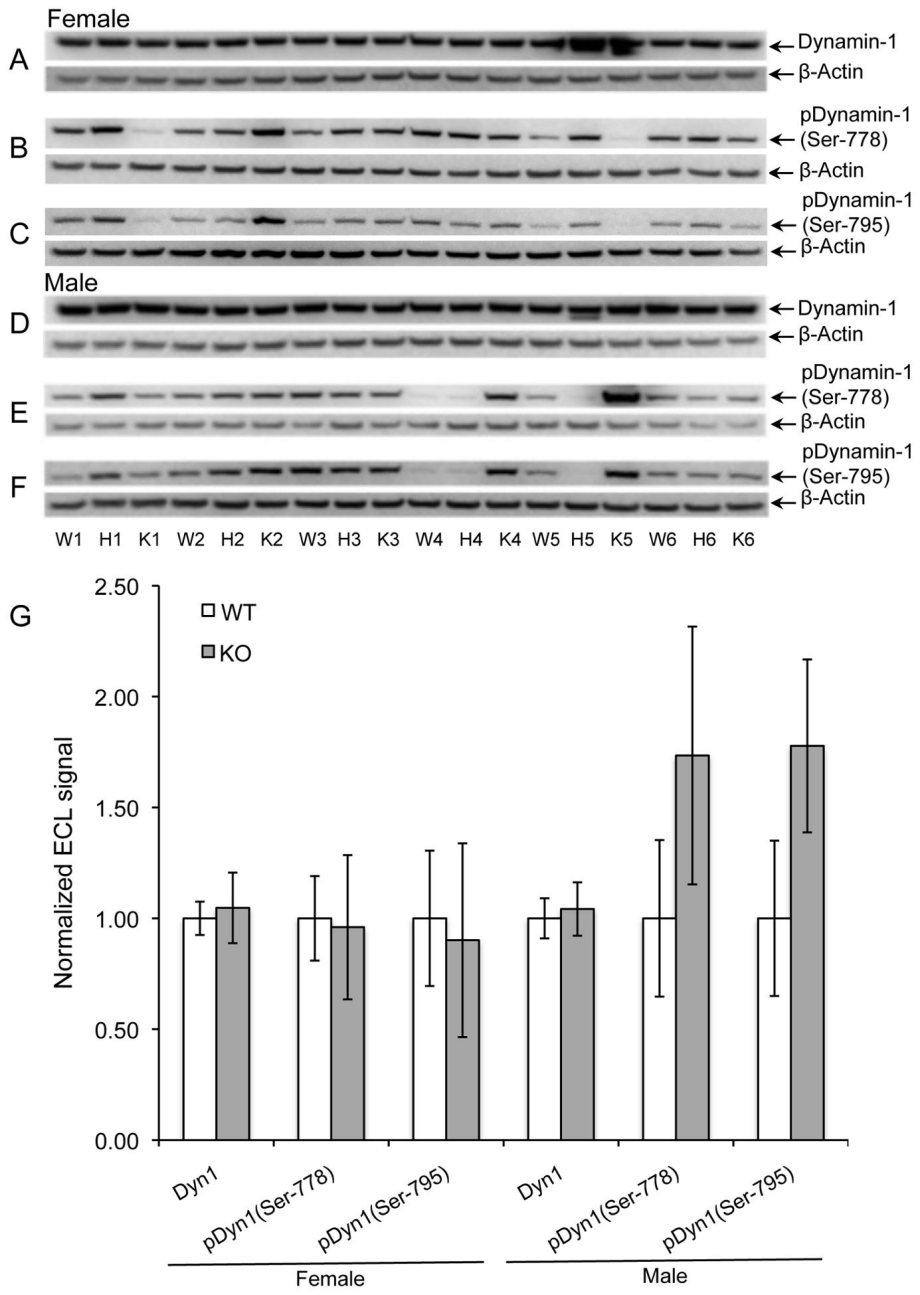
Phosphorylation of dynamin-1 at Ser-778 and Ser-795. Panels A-C show Western blots of brain extracts from female mice for dynamin-1 (A), phosphorylation of dynamin-1 at Ser-778 (B), and phosphorylation of dynamin-1 at Ser-795 (C). Panels D-F: Same as with A-C for male mice. Panel G: Quantitative measurements of band intensities after normalization to β-actin. Data are expressed as means ± SEM (n=5 for each genotype) with statistics as in Figure 1. None of the tests showed a significant difference.

Dynamin-1 is a neuronal GTPase implicated in endocytosis of synaptic vesicles [35] and is another major *in vivo* substrate for PIMT [32]. Dynamin-1 function is regulated by phosphorylation *via* cyclin-dependent protein kinase 1 (Cdk1) at Ser-778, and protein kinase C (PKC) at Ser-795. Figure 4 shows the status of these phosphorylation sites in brain extracts of WT, HZ, and KO mice. The mean of ECL signals for KO males at both phosphorylation sites was increased by 75% over WT males; however the high variance of these signals resulted in a low level of statistical significance (P > 0.05). In contrast, the ECL signal at these phosphorylation sites for female WT and KO mice were nearly identical. No difference in dynamin-1 expression was seen in either sex. Firm conclusions from these results are hampered by the high mouse-to-mouse variability of dynamin-1 phosphorylation, but strongly suggests PIMT deficiency may increase dynamin-1 phosphorylation in males, and not in females. This is an interesting contrast to the results found with the synapsins.

### Phosphorylation of CRMP2 and DARPP-32

CRMP2 plays a key role in axonal guidance during brain development and plasticity of adult brain [36]. It also serves as a major *in vivo* substrate for PIMT [32]. CRMP2 function is regulated by phosphorylation *via* Cdk5 at Ser-522 and by PKC at Ser-795. Western blotting of brain extracts from KO vs. WT mice revealed no effect of PIMT deficiency at either phosphorylation site, or on overall CRMP2 expression, regardless of sex (Figure S1, panels E and F).

We also investigated the expression and phosphorylation of the 32 kDa dopamine and cAMP regulated phosphoprotein (DARPP-32) in KO vs. WT brain extracts. DARPP-32 was of interest because enhanced phosphorylation of synapsin I in KO mice was observed at the cAMP-regulated PKA site, but not at the CaMKII site. Although DARPP-32 is not known to be a substrate for PIMT *in vivo*, it serves as a major target for PKA and, in its phosphorylated state, enhances and prolongs the phosphorylation of other PKA targets by inhibiting their dephosphorylation [37]. If enhanced phosphorylation of synapsin at the Ser-9 site in the KO mice is related to a generalized increase in brain cAMP levels, then one might also see increased phosphorylation of DARPP-32 in the KO mice. As shown in Figure S1 (panels A and B) and Table 3, we saw no significant difference between WT and KO mice in expression or phosphorylation of DARPP-32 in either females or males.

**Table 3:** Summary and statistical analysis of Western blot for female and male wild-type mice.

**Antigen**	N^a^	**Female (WT) signal**	**Female(WT) SE**	**Male (WT) signal**	**Male (WT) SE**	**ttest** ^b^ **P**
PIMT	5	1.00	0.06	1.07	0.07	0.334
Synapsin I	5	1.00	0.11	0.90	0.16	0.352
pSyn Ia(Ser-9)	5	1.00	0.09	0.60	0.04	0.018
pSyn Ib(Ser-9)	5	1.00	0.17	0.61	0.09	0.041
pSyn II(Ser-9)	5	1.00	0.17	0.63	0.14	0.044

^a^Number of animals per genotype

^b^Two-tailed, with matched pairing of adjacent lanes.

### Tubulin Acetylation

**Figure 5:**
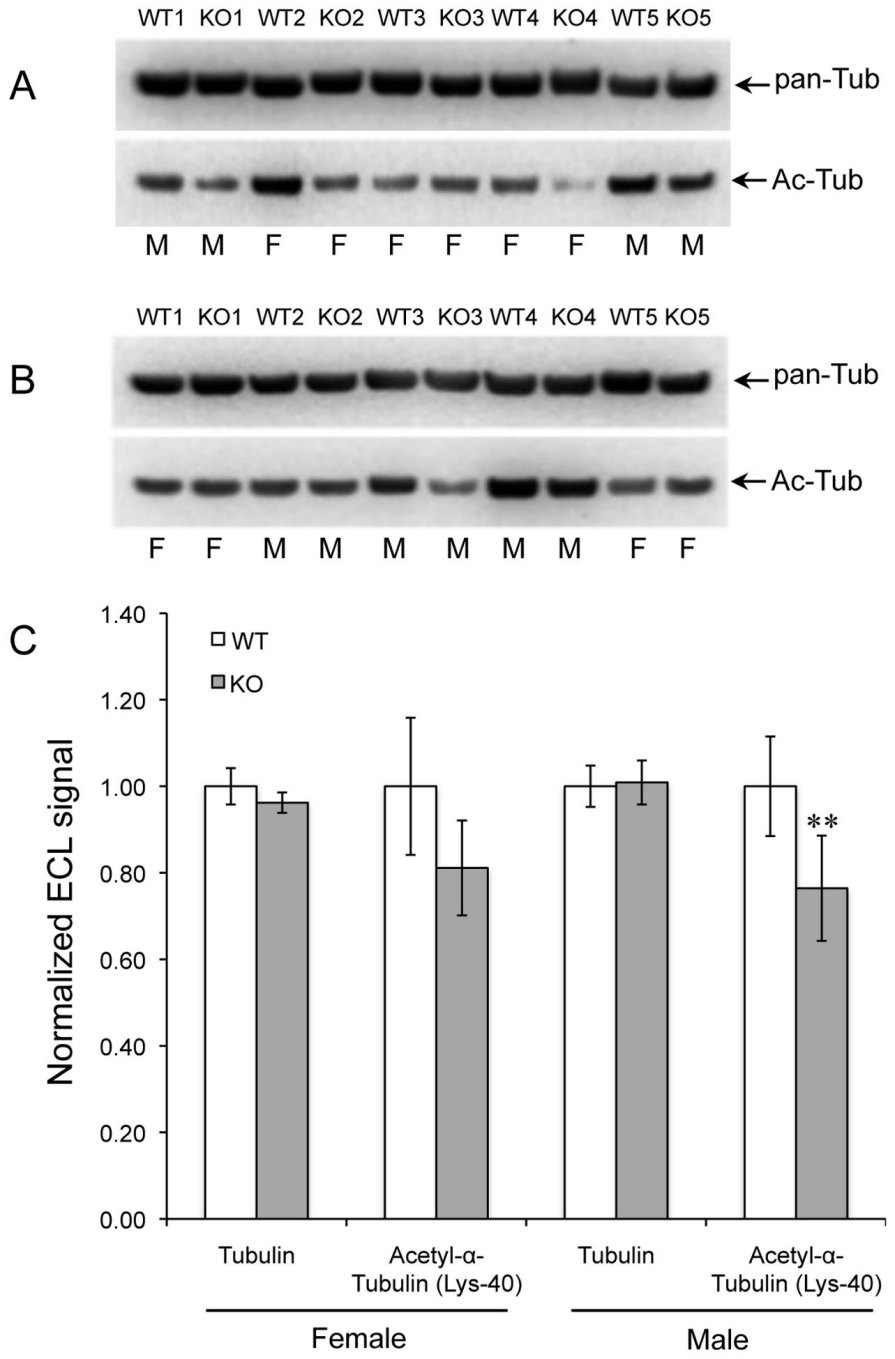
Acetylation of α-tubulin. Western blots for tubulin (pan-Tub) and acetyl-α-tubulin (Ac-Tub) in brain extracts of WT and KO mice, both female and male (A, B). Genotypes are indicated at the top of each lane and sex at the bottom. (C) Quantitative analysis of band intensities for tubulin and acetyl-α-tubulin respectively. Data are expressed as means ± SEM (n=10) with statistics as in Figure 1.

Tubulin is the single largest contributor to isoAsp formation in mouse brain [32,38], and abnormal bundling of microtubules is seen in cortical dendrites of the KO mouse brain [20]. Acetylation of tubulin at Lys-40 is associated with microtubule stability and may also play a role in regulating cell motility and differentiation, intracellular trafficking, and signaling [39]. To see if PIMT deficiency affects the acetylation of α-tubulin, brain extracts from WT and KO mice were subjected to Western blotting with a pan-tubulin antibody, and an antibody specific for tubulin acetylated at lysine-40 (Figure 5A and 5B). Tubulin expression was unaffected by genotype or sex, but acetylation of tubulin was decreased by ca. 20% in both male and female mice (Figure 5C). 

### Phosphorylation of PDK1

**Figure 6:**
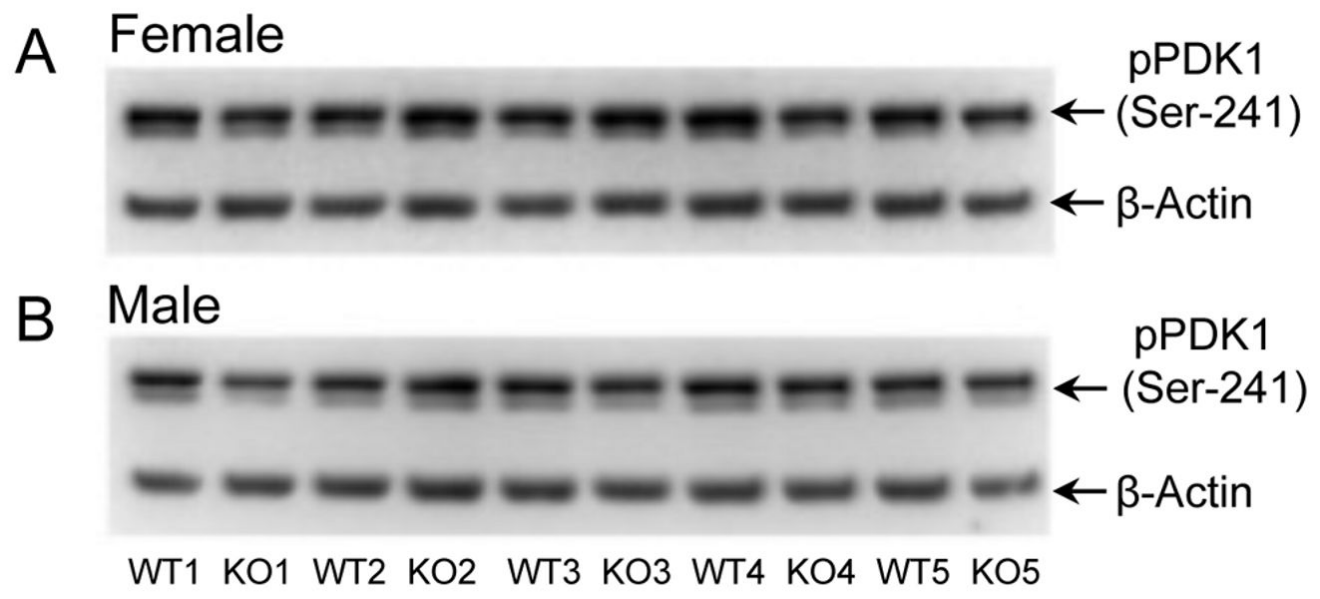
Phosphorylation of PDK1 at Ser-241 is unaffected by PIMT genotype or sex. Western blots showing the phosphorylation of PDK1 at Ser-241 in brain extracts of WT and KO mice, both female and male mice. After normalization of the phospho-PDK1 signals to β-actin, statistical analysis revealed no significant difference between the KO and WT extracts.

PIMT KO mice have enlarged brains, a characteristic also found in mice that have defects in the PI3K/Akt growth signaling pathway. This prompted a study by Farrar et al. [31] who reported increased phosphorylation in PIMT KO vs. WT mouse brain of PDK1 and several other proteins in the insulin signaling pathway. Using extracts of dissected cerebral cortex and hippocampus, these workers reported that phosphorylation of PDK1 in the KO mouse was increased by 260% and 110% respectively, relative to the WT. We attempted to repeat these findings using our own protocol for brain extract preparation and Western blotting (Figure 6). As summarized in Table 2, we were unable to detect any significant difference between WT and KO in either male or female mice. Possible reasons for the discrepancy between our results and those of Farrar et al. are discussed later.

### Overall Protein Phosphorylation Levels are Similar in PIMT KO vs. WT mouse brain.

Attempts to determine if there are overall net changes in protein phosphorylation in the KO mouse brain were carried out in two ways. In the first approach we subjected brain extracts to SDS-PAGE, stained the gel with the phospho-specific protein stain Pro-Q® Diamond, and followed this by staining for total protein with SYPRO Ruby (Figure S2). The ratio of the Pro-Q® Diamond to SYPRO Ruby signals, obtained by image analysis, revealed that there was no significant difference between the WT and KO mice.

In the second approach, we carried out Western blotting of brain extracts from WT and KO mice using a primary antibody (4G10) that is specific for phospho-tyrosine in proteins. After obtaining an ECL image for phospho-tyrosine, the blot was then stained for total protein with Coomassie Blue R-250. (Figure S3). The ratio of ECL to Coomassie signals, obtained by image analysis, revealed that there was no significant difference in overall tyrosine phosphorylation between the WT and KO mice. 

## Discussion

This study was designed to test the hypothesis that specific isoaspartyl-rich proteins in the brains of PIMT-KO mice are functionally altered, and as such, might contribute to the neuropathology characteristic of these mice. Synapsins I and II, α/β-tubulin, dynamin-1, and CRMP2 have been identified previously as major targets for the PIMT repair enzyme in brain as they accumulate exceptional levels of isoAsp sites in the PIMT KO mouse. All four proteins play roles in synaptic function and all are subject to post-translational modifications (PTMs) that regulate those functions. We demonstrate here that *in vivo* phosphorylation of synapsins I and II and acetylation of α-tubulin, are significantly altered in PIMT KO mice, and that dynamin-1 phosphorylation may also be altered. These findings provide a potential basis for the mechanisms by which isoAsp accumulation leads to the neurological deficits characteristic of PIMT deficient mice.

### Synapsin Phosphorylation

Synapsin modulates neurotransmitter release by reversibly anchoring synaptic vesicles to the cytoskeleton in a reserve zone of the pre-synaptic terminal [34]. Increased levels of cyclic-AMP and/or Ca^2+^ promote the release of synapsin from the cytoskeleton, allowing vesicles to migrate to the pre-synaptic active zone. Functional damage to synapsins could make an especially important contribution to the phenotype of the PIMT KO mice, as there are several similarities between the PIMT KO mice and synapsin KO mice. Both types of mutant mice are prone to epileptic seizures [19,40], have cognitive impairment [25,41], exhibit similar neurophysiological abnormalities and display an abnormal distribution of synaptic vesicles in the presynaptic terminal [25,42]

**Figure 7:**
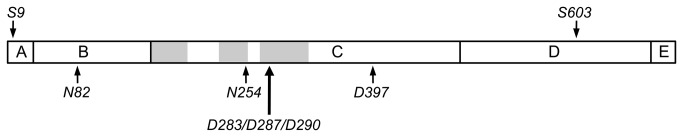
Domain structure of synapsin Ia. Positions of the Ser-9 and Ser-603 phosphorylation sites are shown along the top of the bar. Positions of the major isoAsp sites are shown along the bottom. Shaded areas in domain C indicate the three phospholipid membrane insertion regions.

Purified synapsin aged *in vitro* accumulates isoaspartyl sites at 5-6 residues as indicated in Figure 7 [43]. All but one of these sites is clustered the C-domain, a multifunctional globular region that binds ATP, synaptic vesicles, and several components of the cytoskeleton. Three to four of the C-domain isoAsp sites coincide with sub-regions believed to mediate vesicle binding by insertion into the phospholipid bilayer [44-46]. This coincidence makes it tempting to speculate that isoaspartyl synapsins have a weakened affinity for vesicles. This could promote the redistribution of more synapsin to the cytosol, thereby leading to increased phosphorylation of synapsin at Ser-9, consistent with the phospho-switch model of Hosaka and Südhof [47]. Future experiments may establish the validity of this idea by comparing the affinity of isoaspartyl-rich and isoaspartyl-free forms of recombinant C domain with regard to their relative affinities for synaptic vesicles.

Synapsin phosphorylation has been studied in various contexts for over 30 years, yet (with the exception of animals treated with sex hormones) we are unaware of any prior observations indicating differences between males and females in any animal species. Previous studies on the *in vivo* status of rodent synapsin phosphorylation used rats that were either all male [48,49] or all female [50]. One possible explanation for the dramatic differences we see between male and female brain extracts is that female mice undergo an overall higher level of synaptic activity in response to their handling just prior to, and during, the administration of anesthetic, and that this enhanced neural activity is exaggerated in females. Regardless of the cause, our findings add to a growing list of sex-specific changes in behavior and neurochemistry linked to gene knockouts in mice [51], and add support to the recommendation that possible sex differences in animal models of brain function should be given due consideration in designing experiments [52].

### Tubulin Acetylation

Like the synapsins, α- and β-tubulin are known to be highly susceptible to isoAsp formation *in vitro* [53] and have been shown to be major targets for PIMT in mouse brain [32,38]. Tubulin is an essential element of the cytoskeleton in synaptic regions as well as other parts of neurons. Yamamoto et al. [20] found “deformed apical dendrites” in cortical pyramidal cells of the PIMT-KO mouse that correlated with the presence of “high density bundles” of microtubules not seen in the wild type mouse brain. This suggests that isoaspartyl-damaged tubulin may disrupt synaptic functions that require microtubule integrity. 

The function and detailed mechanism of tubulin acetylation at lysine-40 is poorly understood, but seems to occur either during or after polymerization of α- and β-tubulin to form microtubules [39]. IsoAsp accumulation in tubulin could hinder microtubule assembly, thus explaining the decreased level of acetylation we observed here. It is noteworthy that decreased acetylation of tubulin seen in the KO mice was not significantly different between males and females. This demonstrates that the sex-dependent PTM alterations we observed in the synapsins, and possibly dynamin-1, does not apply to all substrates of PIMT.

### PDK1 Phosphorylation

Farrar et al. [31] compared brain extracts of PIMT KO vs. WT mice with regard to phosphorylation of proteins in the PI3K/Akt growth-signaling pathway because mice with certain defects in this pathway develop enlarged brains, a characteristic also found in the PIMT KO mouse [20]. Using Western blot analysis with phospho-specific antibodies, they found hyperphosphorylation in the KO brains for several proteins in this pathway. The greatest increase was with PDK1 in which the KO mice exhibited an average increase in phosphorylation of 260% and 110%, respectively, in extracts of cortex and hippocampus (P = 0.05 in both cases). In our attempt to repeat this result (Figure 6) we used the same commercial antibody (Table 1) as Farrar, but found no difference between KO and WT. There are three potentially significant differences between our experimental procedures and those of Farrar that, taken together, might contribute to the discrepant results (1). They compared 3 mice of each genotype, whereas we compared 5 mice of each genotype (2). We used mice that were all 28 days old, but they used mice that were 0-50 days old and did not indicate any age matching within a given Western blot (3). We used phosphatase inhibitors in the homogenization buffer and all Western blot solutions [54], whereas they did not indicate the use of phosphatase inhibitors in any of the solutions. The lack of inhibitors, especially in the homogenization buffer, may have allowed unintended dephosphorylation that varied significantly from sample to sample. Combined with the smaller number of mice use, and a possible lack of age matching, the significant difference observed by Farrar may have been a statistical anomaly.

None of the proteins in the current study have any known enzymatic activity, but some of the other PIMT targets identified in our isoAsp proteomic study [32], such as creatine kinase B and HSC70, do have measureable enzyme activity. It will be of interest to see if any of those activities are significantly altered in the KO mouse brain, and if so, are they sex-dependent.

## Supporting Information

Figure S1 **PIMT deficiency does not alter phosphorylation of DARPP-32 at Thr-34, or phosphorylation of CRMP2 at Ser-522 and Thr-514.** *A* and *B*: Western analysis of phosphorylation of DARPP-32 at Thr-34 in brain extracts of female (*A*) and male (*B*) WT, HZ and KO mice. *C* and *D*: Western analysis of phosphorylation of CRMP2 at Ser-522 in brain extracts from female (*C*) and male (*D*) WT, HZ and KO mice. E and F: Western analysis of CRMP2 expression (CRMP2-A/B) and phosphorylation of CRMP2 at Ser-514 (pCRMP2) in brain extracts from female (*E*) and male (*F*) mice.
(TIF)

Figure S2 **Global evaluation of protein phosphorylation in brain extracts of WT and KO PIMT mice by comparing Pro-Q Diamond gel stain for phosphoproteins with the Sypro Ruby general protein stain.** *A*. Gel stained using Pro-Q Diamond dye to detect phosphoproteins.
*B*. The same gel post-stained with Sypro Ruby to visualize total protein. The mouse sex for each lane is indicated at the bottom on panel *A* and *B*.
(TIF)

Figure S3 **Tyrosine phospho-proteome analysis of brain extracts from WT and KO PIMT mice.** *A*, Western blot analysis to visualize the tyrosine-phosphorylated proteins with an anti-phosphotyrosine antibody. *B*, Coomassie Blue stain for total protein on the same membrane. The mouse sex for each lane is indicated at the bottom on panel *A* and *B*.
(TIF)
