# Supplementary figures and images for: Isoaspartate Accumulation in Mouse Brain Is Associated with Altered Patterns of Protein Phosphorylation and Acetylation, Some of Which Are Highly Sex-Dependent
Source: PLoS One. 2013 Nov 5;8(11):e80758. doi: 10.1371/journal.pone.0080758 (PMC3818261; doi:10.1371/journal.pone.0080758)

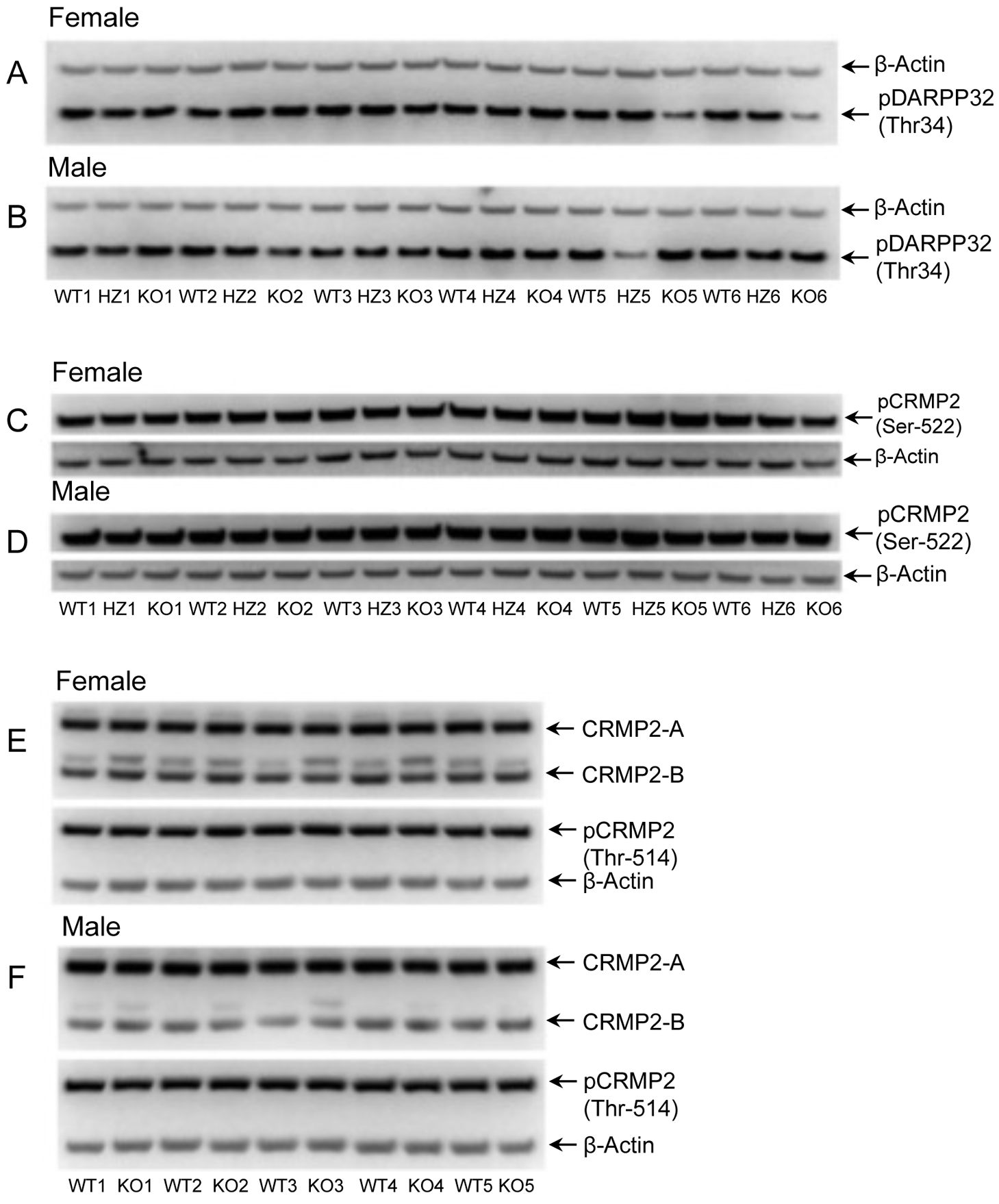

Supplement: Figure S1 — PIMT deficiency does not alter phosphorylation of DARPP-32 at Thr-34, or phosphorylation of CRMP2 at Ser-522 and Thr-514. A and B: Western analysis of phosphorylation of DARPP-32 at Thr-34 in brain extracts of female (A) and male (B) WT, HZ and KO mice. C and D: Western analysis of phosphorylation of CRMP2 at Ser-522 in brain extracts from female (C) and male (D) WT, HZ and KO mice. E and F: Western analysis of CRMP2 expression (CRMP2-A/B) and phosphorylation of CRMP2 at Ser-514 (pCRMP2) in brain extracts from female (E) and male (F) mice. (TIF) [file pone.0080758.s001.tif]

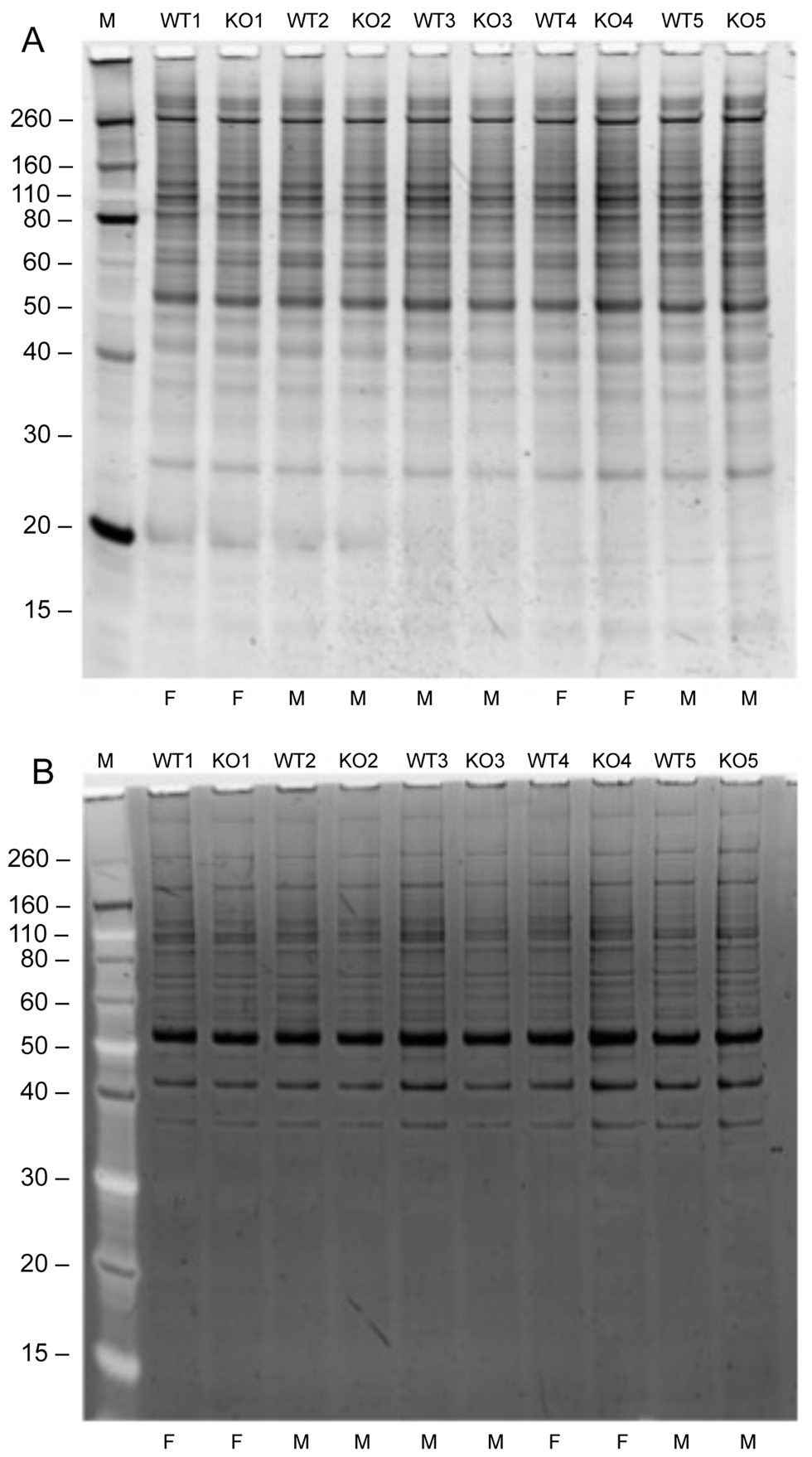

Supplement: Figure S2 — Global evaluation of protein phosphorylation in brain extracts of WT and KO PIMT mice by comparing Pro-Q Diamond gel stain for phosphoproteins with the Sypro Ruby general protein stain. A. Gel stained using Pro-Q Diamond dye to detect phosphoproteins. B. The same gel post-stained with Sypro Ruby to visualize total protein. The mouse sex for each lane is indicated at the bottom on panel A and B. (TIF) [file pone.0080758.s002.tif]

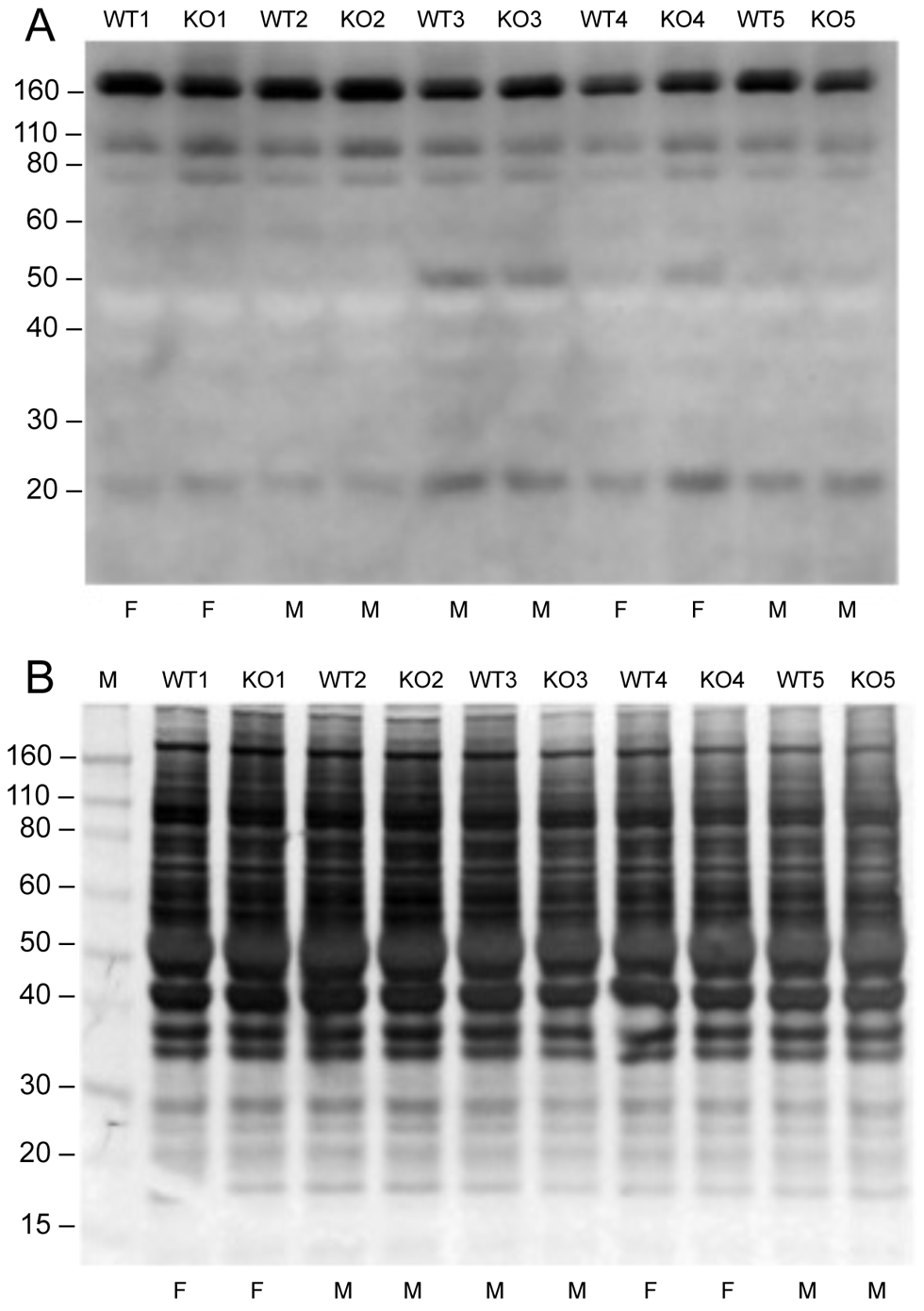

Supplement: Figure S3 — Tyrosine phospho-proteome analysis of brain extracts from WT and KO PIMT mice. A, Western blot analysis to visualize the tyrosine-phosphorylated proteins with an anti-phosphotyrosine antibody. B, Coomassie Blue stain for total protein on the same membrane. The mouse sex for each lane is indicated at the bottom on panel A and B. (TIF) [file pone.0080758.s003.tif]
